# Sortase-mediated immobilization of *Candida antarctica* lipase B (CalB) on graphene oxide; comparison with chemical approach

**DOI:** 10.1016/j.btre.2022.e00733

**Published:** 2022-04-21

**Authors:** Faezeh Moosavi, Faezeh Ahrari, Gholamreza Ahmadian, Mehdi Mohammadi

**Affiliations:** a Bioprocess Engineering Department, Institute of Industrial and Environmental Biotechnology, National Institute of Genetic Engineering and Biotechnology (NIGEB), P. O. Box: 14965/161, Tehran, Iran; b Department of Pure Chemistry, Faculty of Chemistry, Shahid Beheshti University, G.C., Tehran, Iran; c Systems Biotechnology Department, Institute of Industrial and Environmental Biotechnology, National Institute of Genetic Engineering and Biotechnology (NIGEB), Tehran, Iran

**Keywords:** sortase, oriented immobilization, graphene oxide

## Abstract

In this study, *Candida antarctica* lipase B (CalB) was covalently immobilized on the surface of graphene oxide (GO) nanoparticles by sortase-mediated immobilization as well as a chemical attachment approach. Sortase is a transpeptidase that provides one-step purification and targeted immobilization of CalB from one specific site, presenting oriented attachment of the enzyme to a solid support. Chemical immobilization, on the other hand, is considered as a random immobilization, in which the protein can bind to the support from different regions of the protein surface. In this approach, amine-functionalized GO was further modified with glutaraldehyde to facilitate the covalent binding of CalB via its amine residues. The applied methods produced 60% and 100% immobilization yields and presented 0.106 U/mg and 0.085 U/mg of specific activities for the oriented and random immobilization, respectively. The stabilized enzyme with the sortase-mediated approach retained approximately 80% of its initial activity at 50°C.

## Highlights

• Sortase A was used for the oriented immobilization of CalB on graphene oxide nanosheets
• Random attachment of CalB on GO nanosheets were performed by chemical immobilization
• The immobilized CalB were used for the enrichment of omega-3 fatty acids in fish oil
• The derivative obtained from oriented immobilization showed improved selectivity

## Graphical Abstract

**Figure:**
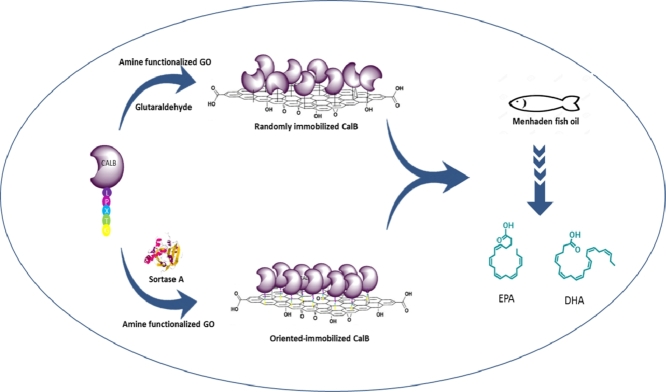

## 1 Introduction

Enzymes are biological macromolecules that act as biocatalyst to increase the rate of reaction by reducing the activation energy. They are generally divided into six categories; oxidoreductase, transferase, hydrolase, lyase, isomerase, and ligase [1]. Lipases (triacylglycerol acyl hydrolase EC 3.1.1.3) with the natural function of catalyzing the hydrolysis of triacylglycerol into glycerol and fatty acids belong to the hydrolase group. They are the most important group of industrial enzymes that are frequently used in several industries including detergents, perfume, food, cosmetics, leather, and synthesis of fine chemicals [2]. Among lipases, *Candida antarctica* Lipase B (CalB) is the most widely used one for the synthesis of organic compounds, showing a high degree of enantio/chemo/regio selectivity [3]. CalB with 317 amino acids and a molecular weight of 33.5 kDa has a secondary structure containing 10 alpha helixes and 9 beta-sheets. The catalytic triad in the active site of this enzyme consists of three amino acids including serine, histidine, and aspartic acid and play a major role in catalysis of lipids hydrolysis [4].

Despite the interesting catalytic properties of lipases, they usually suffer from low stability and limited applicability in their soluble forms. Immobilization of enzymes on a solid support has been proved to be a proper way to improve their functional properties [5, 6]. Immobilization can be divided into 5 main categories: covalent binding, adsorption, entrapment, encapsulation, and cross-linking [7]. Among them, covalent immobilization is the most prevalent method in which the enzyme molecules attach to the surface of support via a strong covalent bond [8]. This type of immobilization is particularly important when the enzyme leaching is a major concern. It also leads to high stability of the immobilized derivatives, improves their applicability and recovery in downstream processing and increases enzyme selectivity in some cases [9]. Moreover, from a different point of view, immobilization can be performed with two general approaches: oriented immobilization and random immobilization. In random immobilization, the enzyme is linked through different sites mostly via parts of the surface rich in lysine residues. In oriented immobilization, on the other hand, the enzyme is attached to the surface only from one specific site [10], [11], [12], [13]. Due to the unpredictable orientation of enzyme in random immobilization, the enzyme attachment may have low reproducibility and can only be used for the cases with relaxed orientation requirements. Furthermore, in this non-specific covalent linkage, the enzyme has the possibility to attach to support via amine groups in the vicinity of active site, thus restricting the accessibility of the active site to the substrate and also preventing easy release of the product to the reaction medium [14, 15]. Therefore, the random immobilization often reduces activity and efficiency of the immobilized enzyme. In oriented immobilization, the enzyme is attached to the surface through a specific site already embedded on the enzyme surface by genetic engineering, providing identical orientation of the protein molecules to the environment [16]. In this way, the target area is selected in a manner to have the maximum distance from the active site. Therefore, in oriented immobilization, it can be expected to have an activity comparable with the activity of the corresponding soluble enzyme. For example, Wang et al. have applied oriented immobilization of enterokinase (EK) on amine-modified GO-magnetic nanosheets via microbial transglutaminase-mediated bioconjugation to improve its stability and reusability [17]. In this approach, a glutamyl donor tag was incorporated into the C-terminal of EK away from the N-terminal active site of the enzyme. The immobilized enzyme effectively retained its intrinsic activity even after several cycles of operation. Vashist et al. were able to immobilize anti-human fetuin A (HFA) antibodies via protein A on the gold (Au) chips. They demonstrated that mass density of the orientated anti-human fetuin A (HFA) antibodies on protein A was higher than the randomly immobilized antibodies [18]. In another study, Mingyang Li et al., were able to site-specifically and covalently immobilize his-tagged proteins via surface vinyl sulfone–imidazole coupling on hydroxyl-terminated self-assembled monolayers (SAMs). They have shown that their method has two advantages of high density and high specificity for protein immobilization [19].

**Scheme 1:**
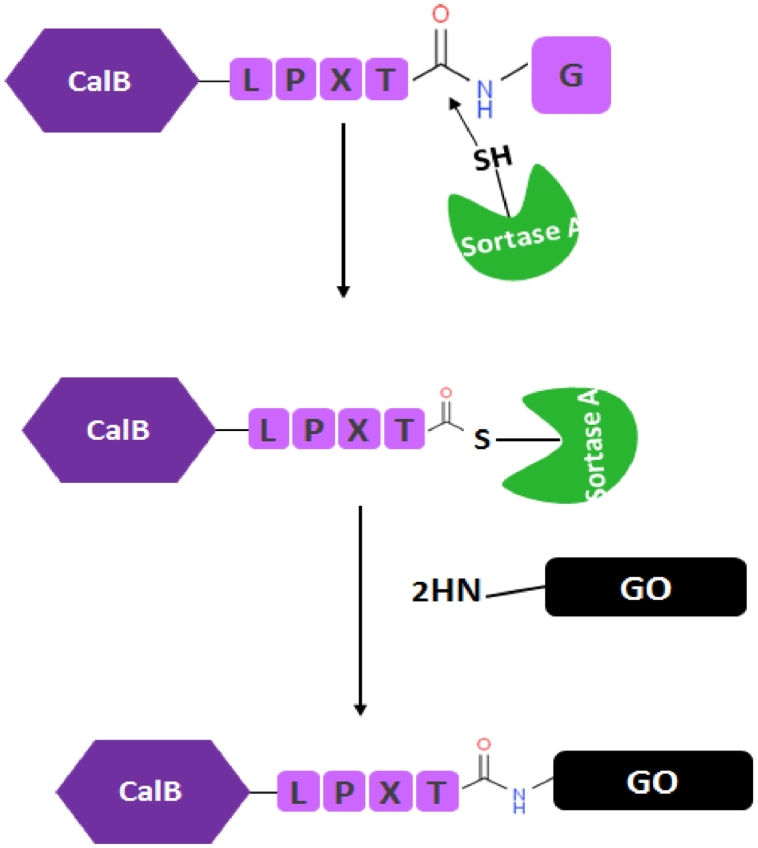
Mechanism of sortase-mediated immobilization

Sortase-mediated immobilization is one of the unique approaches for oriented immobilization. Sortase is a cysteine transpeptidase enzyme encoded in *Staphylococcus aureus*, which can covalently bind its surface protein substrates to the cell wall of gram-positive bacteria [20]. Sortase target proteins have a protected LPXTG motif at the C-terminal. In this motif, X can be replaced by different amino acids (except cysteine) to optimize labeling programs using targeted site-directed mutagenesis. Frequently (including this study) the amino acid glutamic acid, E, is used in the LPXTG motif. Sortase begins its transpeptidase activity by identifying LPXTG amino acid sequences in the target protein. The enzyme then breaks the peptide bond between the amino acid threonine and glycine in the motif and binds to the amino acid threonine of the protein to be stabilized on the surface by the amino acid cysteine at its active site. The binding of sortase to the protein terminates this interaction, by the nucleophilic attack of the free amine group from the matrix, and sortase is released. (Scheme 1) [21].

Sortase-mediated immobilization is a powerful method for covalent attachment of enzymes on solid supports [22]. For the first time, Parthasarathy et al. have used sortase to immobilize LPETG-labeled eGFP (enhanced green fluorescent protein) to (Gly)3- conjugated polystyrene matrix. It was also shown that eGFP binds to (Gly)3 on the support surface even in its impure form, indicating sortase specificity for LPXTG-labeled substrates [23]. Qafari et al, have used sortase A transpeptidation specificity for oriented attachment of protein A as a natural LPXTG-containing sortase substrate, thereby developing an immunoadsorbent using a single-step immobilization method [24]. They modified the surface of silica and graphene oxide (GO) nanosheets with a set of linkers as nucleophile donor groups including (glycine)5–NH2 and –(CH2–CH2)–NH2, to address the effects of various nucleophiles and supports on the functional properties of the immobilized protein. Using a functional IgG purification assay, they found that ethylenediamine on the surface of GO was more effective than pentaglycine as a native nucleophile donor, which is widely used for sortase-mediated immobilization.

Using the sortase-mediated technique, Hata et al. were able to immobilize Thermobifida fusca β-glucosidase and Streptococcus bovis α-amylase on the surface of (Gly)3-conjugated polystyrene nanoparticle [25]. They investigated the activity, stability, and reusability of the proteins C-terminally fused with the sortase recognition motifs. Both immobilized enzymes retained their stability and catalytic activity and showed a 3.0 and 1.5-fold increase in activity compared to chemically bonded enzymes, respectively. Ito et al. proposed an efficient method for the immobilization of recombinant glycosyltransferases onto sepharose by means of transpeptidase reaction by sortase A. They indicated that site-specifically immobilized enzymes exhibited the desired sugar transfer activity, improved stability, and practical reusability required for rapid and large-scale synthesis of glycoconjugates [25].

In this study, a most widely used lipase of CalB was immobilized on the surface of graphene oxide as a support using two different approaches of oriented or random immobilization. For oriented immobilization, the conserved sortase recognition motif was genetically fused to the C-terminal region of CalB. Using this strategy, the LPXTG motif on CalB was identified by sortase, the peptide bond between the threonine and glycine was cleaved and CalB was covalently attached onto the surface-modified GO nanosheets in a specific direction. For random immobilization, the enzyme was chemically attached by glutaraldehyde treatment to the GO surface randomly in different directions. The performance of the two methods was compared.

## 2 Material and method

### 2.1 Materials

The fish oil from menhaden (containing 10-15% of eicosapentaenoic acid and 8-15% of docosahexaenoic acid), cis-4,7,10,13,16,19-docosahexaenoic acid, Tris base, ethylenediamine and N-(3-dimethylaminopropyl)-N-ethylcarbodiimide hydrochloride (EDC) were from Sigma. Glycine was from Carl Roth. Graphite (95.5%, 2-18 nm with 32 lyers) was from US research Nanomaterials Inc, USA. Other reagent and solvent were from Merck. Scanning electron microscope (SEM) analysis were recorded on a TESCAN, VGA3 (Tescan, Czech Republic) operating 20 kV accelerated voltage at ambient temperature. Thermogravimetry analysis (TGA) was carried out from 10°C to 800°C at a heating rate of 10°C min−1 in air atmosphere via Q600 system TA company, USA. Fourier transform infrared spectra (FT-IR) were recorded on a Bomen FT-IR-MB-series instrument with KBr as a matrix.

### 2.2 Methods

#### 2.2.1 Construction of the recombinant pET-calB plasmid and transformation

The gene encoding of CalB was synthesized by GenScript and inserted in the vector pET-26b (+) between the two restriction sites NdeI and XhoI. The recombinant plasmid, called pET-calB, was then transformed into E. coli BL21 (DE3) using heat shock method previously described in the literature [26] and screened for the presence of the recombinant strains on LB-Ampicillin medium. The LPXTG sortase cleavage motif and the 6 x histidine tag were inserted at the C-terminal end of the CalB before the stop codon, respectively. The cleavage motif was separated from histidine tag by a rigid linker (AEAAAKEA) to prevent steric hindrance.

For the expression of sortase, a recombinant plasmid construct was used that contained the coding region of the *S. aureus* sortase (srtA), lacking its first 60 amino acids of N-terminal region and contained a histidine tag at its C-terminal region, previously cloned into the pQE30 vector [24].

#### 2.2.2 Expression and purification

The recombinant *E. coli* BL21(DE3) cells harboring the recombinant vector were pre-cultured in LB medium (5 g/L yeast extract, 10 g/L trypton and 10 g/L NaCl) containing kanamycin 50 mg/ml at 37°C and 180 rpm for 16 h. It was then inoculated as 1% into the new LB medium contains kanamycin 50 mg/ml at 37°C and 180 rpm. After reaching the appropriate OD600 to 1, the protein expression was induced using 1 mM of IPTG at the final concentration and incubated at 30°C and 100 rpm for 16 h. The bacterial cells were lysed by sonication on ice 6 times each for 1 minute with a 45 seconds interval at a frequency of 70 kHz. The recombinant CalB enzyme was purified by affinity chromatography using Ni-NTA beads (Qiagen, USA) as it contains a histidine-tag at its N-terminal. The purified protein prep was then dialyzed in 25 mM phosphate buffer and stored at 4°C. Expression of the recombinant protein was confirmed by SDS-PAGE analysis. Protein concentrations were measured by the Bradford method [27].

#### 2.2.3 Activity measurement and calculation of the immobilization yields

Activity of the soluble and immobilized derivatives was spectroscopically determined by hydrolysis of *p*-nitrophenyl butyrate (*p*-NPB) as a substrate [28]. The lipase-catalyzed hydrolysis of 0.8 mM *p*-NPB in a 10 mM sodium phosphate buffer (pH 8, 25°C) solution produced *p*-nitrophenol which was detected at 410 nm. The activity recovery for both oriented and random immobilization was calculated as the ratio of the activity of immobilized CalB to the activity of the same amount of soluble CalB. The amount of immobilized RML was calculated by subtracting the amount of the enzyme in the supernatant from the total amount of the lipase used for immobilization. The immobilization yields were determined as the ratio of the amount of CalB on the support to the initial amount of the protein in the solution and reported as percentage.

#### 2.2.4 Graphene oxide (GO) preparation

Graphene oxide (GO) nanosheets were prepared from graphite as follow: The suspension of graphite (0.5 g) in a 50 ml of 98% sulfuric acid solution was vigorously stirred in an ice bath for 10 minutes. Then potassium permanganate (3 g) and sodium nitrate (0.5 g) were gradually added to the mixture over 2 hours. The suspension was then warmed up to room temperature followed by adding 100 ml of deionized water. Then the reaction was performed for 2h at 98°C. After decreasing the temperature to 60°C, 3 ml of H_2_O_2_ (30%) was added to the solution. The solid product was then separated by filtration, washed thoroughly with 10% hydrochloric acid and then deionized water. Finally, the solid particles were sonicated for 20 minutes in a probe sonicator, freeze dried and stored at 4°C.

#### 2.2.5 Chemical amination of GO nanosheets

The dry GO particles (10 mg) were dispersed in 2 mL phosphate buffer solution (10 mM, pH 4.8) containing ethylenediamine (0.06 M) followed by adding EDC in final concentration of 10 mM. The resulting mixture was stirred for 3 h, washed with deionized water, centrifuged and stored at 4°C.

#### 2.2.6 Chemical immobilization of CalB on GO-NH_2_

GO-NH_2_ (2 mg) was added to 1 ml solution of 200 mM phosphate buffer pH 7 containing glutaraldehyde (5% w/v) and stirred under magnetic stirrer at room temperature for 4 h. After centrifugation at 5000 rpm, the glutaraldehyde-modified particles were washed with 25 mM phosphate buffer pH 7 and re-suspended in 1 ml of the same buffer and stored at 4°C. For immobilization of CalB, 2 mg of the modified support was added to 1.5 ml of the purified enzyme (0.17 mg/ml) in 25 mM phosphate buffer (pH 7) and stirred at room temperature. After 24 h, the immobilized preparation was centrifuged, dissolved in the appropriate amount of phosphate-buffered saline (PBS) and stored at 4°C. The supernatant was analyzed to confirm immobilization by both activity assay and protein concentration determination by the Bradford method.

#### 2.2.7 Oriented immobilization of CalB on GO-NH2 by using sortase

The prepared GO-NH2 (10 mg) together with 40 μl of soratase and 800 μl of sonicate supernatant was added to 80 μl of soratase buffer 10X (containing 1.5 M sodium chloride, 0.5 M tris acid, 75 mM calcium chloride pH 7.5) and the reaction was stirred overnight at 40°C. The immobilized CalB was then centrifuged, dispersed in 1 ml of 25 mM phosphate buffer and stored at 4°C. A control experiment was conducted in the same condition without using SrtA.

#### 2.2.8 Thermal stability of the immobilized derivatives

The effect of temperature was determined at various temperatures ranging from 40°C to 55°C to evaluate the thermal stability of the stabilized enzyme. The samples were exposed to each temperature for 2 h and then subjected to enzyme activity assay using *p*-NPB assay to determine the residual activity according following equation:

$$residual\;activity\left(\%\right)=\frac{activity\;after\;incubation}{initial\;activity\;at\;25^{\circ }C}*100$$

#### 2.2.9 pH optimization analysis

The effect of pH on enzyme activity was determined in 10 mM sodium phosphate buffers in different pH values ranging from 4.5 to 9.5. The *p*-NPB assay was used to determine the activity of each sample.

#### 2.2.10 Stability of the immobilized preparations in the present of co-solvents

To investigate the effect of organic solvents on the activity of each lipase derivative, they were suspended in a solution containing 20% of methanol, ethanol and propanol for 16 h. The *p*-NPB assay was used to determine the activity of each sample.

#### 2.2.11 Enzymatic hydrolysis of fish oil

The lipase-catalyzed hydrolysis of fish oil was performed in an organic-aqueous biphasic system [13]. 1.5 mL of cyclohexane, 150 µL of fish oil and 1.5 mL of phosphate buffer (25 mM) pH 7.0 were added in a tube and pre-incubated for 15 min with vigorous stirring at 25°C. The reaction was then initiated by adding 5 mg of different immobilized derivatives. The concentration of free fatty acids during of the reaction progress was determined by taking 100 µL of organic phase at selected time intervals followed by addition of 200 µL of 2-propanol. The results were then evaluated by using the reverse-phase HPLC (Knauer equipped with an UV detector) with a C18 column (25 cm × 0.46 cm). The mobile phase was 67% of acetonitrile, 33% of water and 0.5% acetic acid at flow rate of 0.7 ml/min and 210 nm in the UV detector. The retention times for the unsaturated fatty acids were 17 and 19 min for EPA and DHA, respectively. Experiments were conducted in triplicate, and the relative errors never exceeded 5 %.

## 3 Result and discussion

### 3.1 Preparation of the modified GO nanosheets

SrtA naturally has high specificity for the multiglycines as free amine donor substrates. However, good performance of SrtA in enzyme immobilization has been reported for the amine group bonded to at least one sp^3^ carbon atom (-CH_2_-NH_2_) [24].

In our previous study, we also demonstrated the efficiency of ethylenediamine to use for functionalization of solid supports and subsequent use of the functionalized supports as an amine donor group in sortase-mediated immobilization of protein A [24]. To provide similar condition for sortase-mediated immobilization of CalB, the amine group was introduced to the surface of GO nanosheets by the reaction of carboxylic acids group of GO surface with ethylenediamine.

### 3.2 Expression of Sortase A ∆59 and CalB

**Fig. 1:**
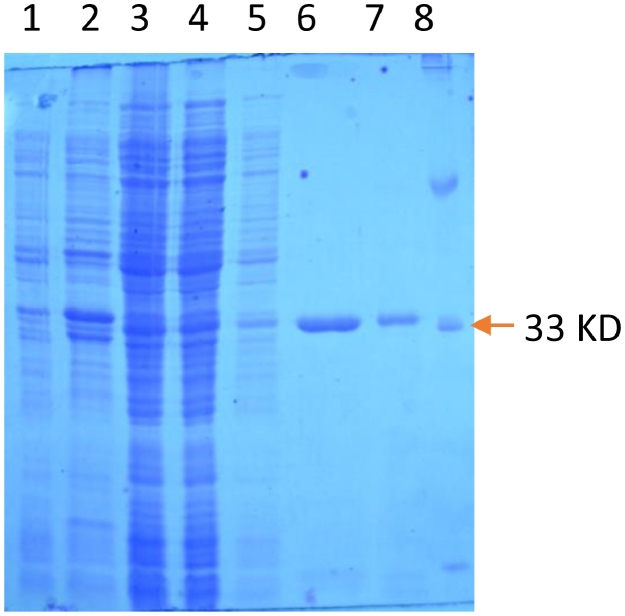
Expression and purification of recombinant CalB, Lane 1: Negative control for expression, Lane 2: Sediment after sonication, Lane 3: Supernatant after sonication, Lane 4: Flow-through after binding to Ni-NTA, Lane 5: Wash sample, Lane 6, 7: Purified CalB, Lane 8: protein marker.

SrtA∆59 was produced by cloning of SrtA gene from the *Staphylococcus aureus* (ATCC35556D) into the pQE-30 plasmids. This construct contains a truncated form of the *Staphylococcus aureus* sortase, in which the first 59 amino acids in the N-terminal region have been deleted. Recombinant sortase A containing 6X His-tag was expressed in *E. coli* BL21 DE3, and then purified by affinity chromatography which was subsequently confirmed by SDS-PAGE analysis. The lipase used in this study has previously been described [26]. The construct consists of the lipase coding region, which is located downstream of a sec-dependent signal sequence, followed by the sortase cleavage motif, followed by the 6X His-tag at the end of C-terminal. A rigid linker including S R S A E A A A K E A is located between the sortase cleavage motif and the 6x His-tag amino acids to ensure that the different domains are well separated and to prevent interference with sortase activity. For the expression and purification of CalB, plasmid pET-26-CalB was transformed into the *E. coli* BL21 DE3. The expressed recombinant lipase was then purified by affinity chromatography and finally analyzed by SDS-PAGE analysis (Fig. 1).

### 3.3 Immobilization of CalB

**Fig. 2:**
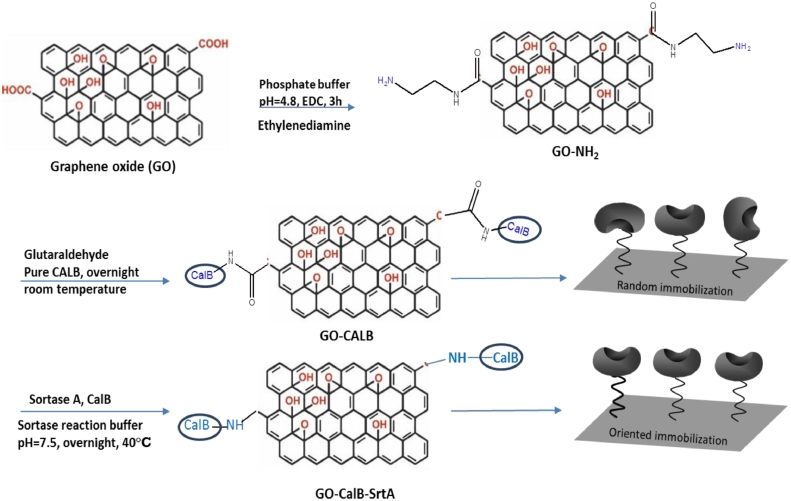
A schematic illustration of oriented and random immobilization of CalB on modified GO nanosheets

In order to investigate the effect of different immobilization protocols on the functional properties of the immobilized derivatives, CalB was covalently immobilized on the modified supports via both the chemical and enzymatic approaches (Fig. 2). Chemical attachment to the glutaraldehyde-modified support and enzymatic immobilization of CalB represent random and oriented immobilization, respectively.

The lipase used for the immobilization was genetically engineered to have an LPxTG domain on its external surface and used for enzymatic immobilization after the protein expression without any further modification. To this, the expressed CalB was directly immobilized on GO nanosheets after bacterial cells were broken down and the cell debris were removed. In this approach, threonine residue of LPxTG motif is used for immobilization, meaning that the lipase immobilization occurs from its C-terminal. However, there are some other studies that confirm the application of a same approach for covalent attachment of enzymes via their N-terminal [29]. Enzymatic immobilization of CalB was performed as shown in Fig. 2 at 40°C and pH 7.5 as an optimal condition for SrtA activity. The enzyme activity assay showed that almost 60% of CalB was immobilized on the support after 16h of incubation producing a specific activity of 0.106 U/mg. This means that sortase-mediated ligation also provides a strategy for one-step purification and simultaneous immobilization of CalB exclusively through the LPXTG domain. High activity recovery (132%) was obtained for the immobilized CalB most likely because of the proper and uniform orientation of the enzyme molecules on the support that facilitates accessibility of the substrate to the enzyme active site. The obtained result is very interesting once compared to the traditional nonspecific chemical immobilization, in which the target enzyme must be purified before immobilization to achieve higher protein loading and volumetric activity.

In parallel with the ongoing researches in genetic engineering and enzyme immobilization, many studies have tried to take advantage of this approach to combine enzyme immobilization with its purification [30]. For example, already immobilized monoclonal or polyclonal antibodies have been used for the selective immobilization of proteins on a variety of solid supports [31]. Recently, solid heterofunctional supports have been reported as a promising tool to couple purification and immobilization processes. In this approach, one functional group initially adsorbs the target protein from a crude lysate then the second functional group facilitates the covalent attachment of the pre-adsorbed protein [32].

In the other approach, chemical immobilization of the purified CalB was performed on amine-functionalized GO in the presence of glutaraldehyde at pH7. This random immobilization occurs mostly via amine groups of lysine residues on the enzyme surface. The surface exposed amine groups of the enzyme reacts with the aldehyde groups of glutaraldehyde which as a cross-linker has a same reaction with the amine groups of the support in a same manner. The results showed 100% immobilization yield after 24h incubation of the enzyme. The specific activity of the immobilized biocatalyst was 0.085 U/mg that is 20% lower than the specific activity of the immobilized derivative obtained from oriented immobilization by sortase. In fact, chemical immobilization as a nonspecific process proceeds via different sites of the protein surface and those linkages at or near the active site can damage the activity of the immobilized protein. This was proved by much lower activity recovery (63%) compared to the activity recovery of oriented-immobilized CalB. Moreover, higher immobilization yield in the chemical approach compared to the enzymatic immobilization probably causes to increase the diffusion limitation of the substrate to the active site of the immobilized enzyme.

### 3.4 Characterization of the support before and after immobilization

**Fig. 3:**
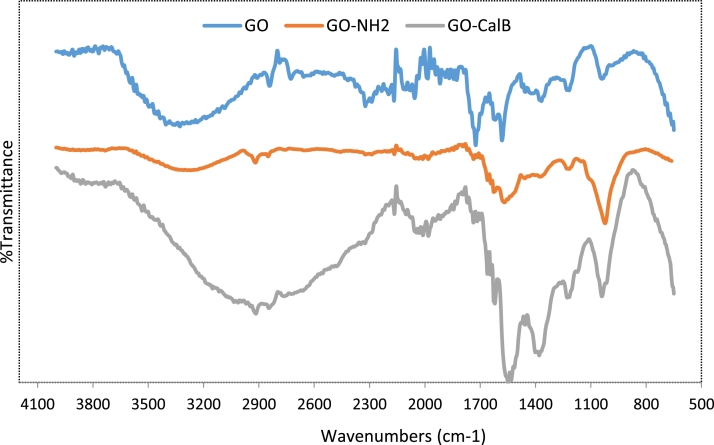
FT-IR spectra of GO derivatives

The thermal gravimetric analysis (TGA), scanning electron microscopy (SEM) and FT-IR spectroscopy were used to confirm the successful preparation of the support. The IR spectrum of GO nanosheets showed successful oxidation of graphite (Fig. 3). The broad and intense peak centered at 3404 cm^−1^ can be attributed to the O-H groups of GO surface. The C=O stretching vibrations of the carboxylic functional group can be observed at 1724 cm^−1^ as a sharp peak.

The two absorption peaks at 1218 cm^−1^ and 1031 cm^−1^ were attributed to the C-O stretching vibrations of the epoxide groups. After amination almost a same pattern was observed with diminished intensity of the band related to carboxylic acid groups, confirming a decrease in number of carboxylic acids due to reaction with amine groups of ethylenediamine. The immobilized derivative also presented more broad and intensive peak for carboxylic acid groups between 2200-3600 cm^−1^ due to high number of these functional groups on the surface of the enzyme, showing that the enzyme molecules are attached to the surface of GO nanosheets.

**Fig. 4:**
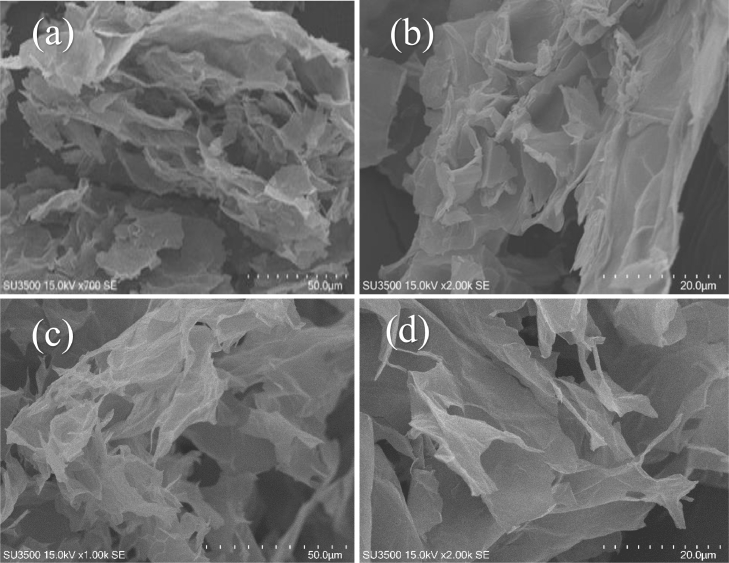
Scanning Electron Micrograph (SEM) images of graphene oxide (a, b) and (c, d) GO-CalB-SrtA

SEM imaging was conducted to evaluate the morphology of GO nanosheets before and after enzyme immobilization. The bare GO particles (Fig. 4 (a,b)) presents nanosheets with a lot of wrinkles on their surface that make it a proper support bearing high surface to volume ratio for efficient enzyme immobilization. Different surface morphology was observed after immobilization (Fig. 4 (c,d)), in which the smoother surface of GO nanosheets after enzyme immobilization shows a uniform mass covering of the sheets and confirms that the support is evenly filled by the lipase.

**Fig. 5:**
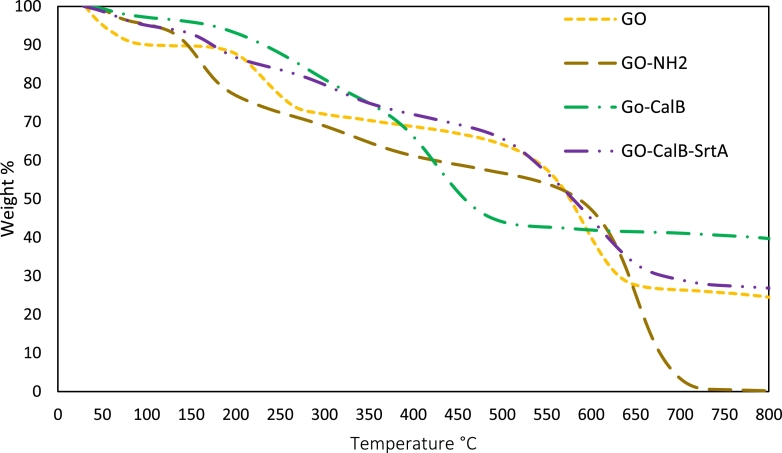
Thermal gravimetric analysis of the derivatives obtained from GO nanosheets

To further prove the successful functionalization /immobilization of GO in each step and to have more insight in thermal stability of the prepared derivatives as well, TG analysis was performed and the results compared with GO. Fig. 5 shows TG curves of GO, amine functionalized GO (GO-NH_2_), chemically immobilized CalB (GO-CalB) and sortase-assisted immobilized CalB (GO-CalB-SrtA) in the temperature from 30°C to 800°C. All the samples showed a mass loss of up to 5-10% during the initial heating up to 110°C, which corresponds to the removal of free water molecules from the derivatives. Two other thermal events can be observed in the TG curves of all the samples, indicating a considerable mass loss during a continuous increase in temperature.

The second mass reduction in GO (21.2%) occurred between 110 to 403°C, which could be associated with the decarboxylation process of the graphene oxide sheets [33]. A mass loss of 34.6% was observed for amine-functionalized GO between 106°C to 411°C due to the removal of chemically added amine groups from the surface. The higher degree of the observed mass loss (13.3%) compared to the bare GO in the same range of temperature, presents successful introduction of amine groups on GO sheets. This became even more pronounced after glutaraldehyde-assisted immobilization of CalB, where the related curve showed a mass loss of 51.5% in the temperature range from 106°C to 528°C. For the derivative obtained from immobilization by sortase (GO-CalB-SrtA), a mass loss of 28.5% was observed in the same temperature range, meaning that the enzymatic immobilization produces a derivative with a higher thermal stability compared to GO-CalB. However, both derivatives showed almost the same thermal behavior up to 410°C, showing a mass loss of about 25%. For all curves, the range of 530 to 800°C corresponds to the decomposition of the more stable functional groups such as epoxy and carboxylic acid groups [34].

### 3.5 Stability of the immobilized derivatives

**Fig. 6:**
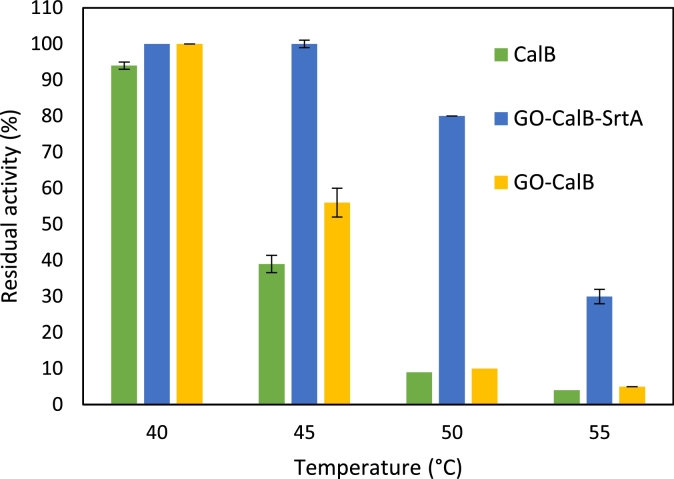
Thermal stability of the soluble and immobilized derivatives of CalB. Experimental condition: Incubation of 1mL sodium phosphate buffer 25 mM (pH 7.0) containing 1-2 mg of each biocatalyst at different temperatures for 2h. The initial activity for each derivative was determined in 1 mL of 25 mM sodium phosphate buffer (pH 7.0) at 25°C before incubation and set as 100%. CalB in its soluble form was obtained by purification of the expressed enzyme from the crude by using affinity chromatography

Thermal stability of the immobilized preparations obtained from random (GO-CalB) and oriented (GO-CalB- SrtA) immobilization was evaluated at different temperatures (40°C -55°C) and the results were compared to thermal stability of the soluble enzyme. Fig. 6 presents the remaining activity of the immobilized and soluble CalB after 2h of incubation in each temperature.

Both immobilized derivatives retained 100% of their activities at 40°C which was almost similar to the thermal stability of the soluble CalB that lost only 6% of its initial activity under the same condition. With increasing the temperature to 45°C, while the sortase-assisted immobilized CalB remained completely stable, the soluble enzyme and GO-CalB lost 44% and 61% of their initial activities, respectively. The greater stability of the CalB derivative obtained from oriented immobilization compared to GO-CalB, is most likely because of the deleterious effect of the enzyme-support linkages formed in the vicinity of the enzyme active site in the random immobilization approach. Significant difference was observed in the thermal stability of the derivatives at 50°C, where GO-CalB-SrtA retained 80% of its initial activity while GO-CalB and soluble CalB lost almost 90% of their activities, further confirming the positive effect of the oriented immobilization on the thermal stability of the enzyme. Further increase in temperature to 55°C significantly reduced the residual activity of GO-CalB-SrtA and GO-CalB by 30% and 5%, respectively. The effect of oriented immobilization on protein stabilization compared with derivatives obtained in random immobilization has already been reported in other studies [35]. .

**Fig. 7:**
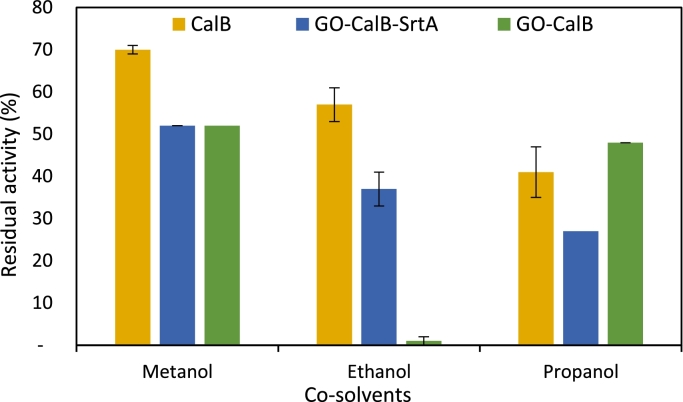
The effect of organic solvents on activity of soluble and immobilized CalB, experimental condition: Exposing CalB and immobilized forms into 1 mL of sodium phosphate buffer (25 mM, pH 7.0) containing 20% (v/v) of methanol, ethanol and propanol at 25°C for 16h. The initial activity of CalB and immobilized derivatives determined were determined in 1 mL of 25 mM sodium phosphate buffer (pH 7.0) before incubation in the presence of co-solvents and set as 100%.

The possibility of performing biocatalytic reactions in the presence of organic solvents has been found to be useful tool for the application of biocatalysts [36]. Three polar organic solvents (20%) were used to investigate the co-solvent stability of soluble CalB, GO-CalB-SrtA and GO-CalB (Fig. 7). The results showed that 16 h incubation in the presence of methanol as the most polar solvent, led to a 48% decrease in initial activity of GO-CalB-SrtA and GO-CalB while the soluble enzyme retained 70% of its activity in the same condition. Changing the solvent to ethanol caused to complete deactivation of GO-CalB, whereas the soluble CalB and GO-CalB-SrtA remained 57% and 37% active, respectively. Propanol was the only solvent in the presence of which, GO-CalB remained more stable compared to the soluble enzyme while retaining 48% of its initial activity. The results suggest that immobilization of CalB via both random and oriented immobilization had no meaningful effect on their co-solvent stability.

### 3.6 pH optimization

**Fig. 8:**
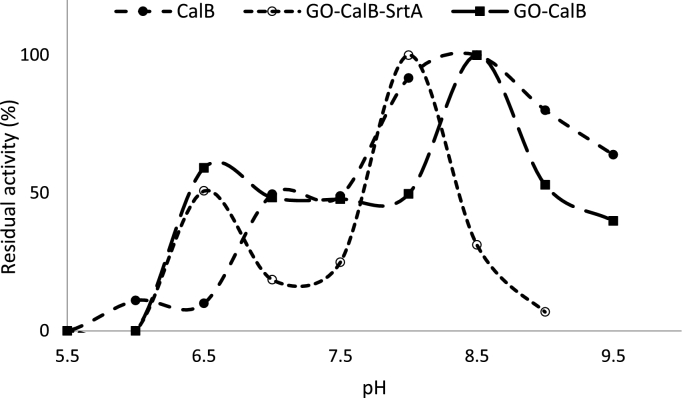
Optimum pH activity of the immobilized derivatives CalB

The optimization study on the effect of pH on activity of the soluble and immobilized lipase derivatives was conducted by measuring the activity of enzyme samples in solutions with pH values ranging from 5 to 10 at 25°C, the results of which are shown in Fig. 8. The optimum pH values were determined to be 8.0 for GO-CalB-SrtA and 8.5 for soluble CalB and GO-CalB. Altering the electrostatic charge of the enzyme after immobilization can be considered as the main reason for changing optimum pH activity of GO-CalB-SrtA [37]. There are similar examples in the literature reporting different optimum pH for immobilized enzymes compared to their corresponding soluble forms [38]. The optimal pH shifting towards acidic value of immobilized enzyme have been similarly reported by Ladole et al. after immobilization [39]. Furthermore, the activity assay of each biocatalyst over a range of pH revealed an interesting bimodal distribution of activity at the pH range examined.

Barboza-Corona, et al. have attributed this phenomenon to the presence of two conformational arrangements of the enzyme molecule inside the cell, which present unusual activity at different pHs [40].

### 3.7 Selectivity of the immobilized derivatives

The performance of lipases in producing cis-5, 8, 11, 14, 17-eicosapentaenoic acid (EPA) and cis-4, 7, 10, 13, 16, 19-docosahexaenoic acid (DHA) from the selective hydrolysis of fish oils has been reported in several studies. EPA and DHA, as two omega-3 fatty acids with almost similar physical properties are the most essential omega-3 polyunsaturated fatty acids (PUFAs) for human health. We performed the hydrolysis of fish oil from menhaden to investigate the effect of the random and oriented immobilization of CalB on the lipase activity and more importantly on selectivity of the immobilized derivatives of CalB. The hydrolysis reaction was performed at 4°C and 30°C in pH 7 in a biphasic system. The results showed that the activity of both derivatives reduced with decreasing the temperature to 4°C. GO-CalB-SrtA also showed higher activity in the fish oil hydrolysis compared to the chemically immobilized CalB. This is in accordance with the result obtained from the activity assay performed by p-NPB as a small substrate. The results of selectivity showed that both immobilized CalB discriminated between EPA and DHA in favor of EPA at both temperatures (Table 1). This means that EPA ester bond is more easily hydrolyzed compared to DHA ester bond. This can be more likely attributed to differences in the structures of EPA and DHA that have effective influence on substrate acceptability of CalB independent to the reaction temperature. DHA has a double bond at 4-position which is closer to the site of hydrolysis compared EPA having a double bond at 5-position [41]. It has also been reported that the enzyme selectivity might be affected by immobilization due to some other reasons such as changing diffusion limitation after immobilization, altering KM or Kcat values of the enzyme towards the substrate and generation of new micro-environments around the enzyme [42]. Furthermore, GO-CalB showed almost the same selectivity in both temperatures, while GO-CalB-SrtA showed higher selectivity when the rate of the hydrolysis reaction decreased by lowering the temperature to 4°C. The most interesting result obtained in the hydrolysis reaction performed by GO-CalB-SrtA at 4°C, producing the selectivity of 10.7 which was almost 3.2 fold higher than the selectivity of GO-CalB in the same condition. The improved selectivity of GO-CalB-SrtA vividly confirmed the positive effect of oriented immobilization of CalB on its functional properties.

**Table 1:** Activity and selectivity of GO-CalB and GO-CalB-SrtA at pH 7

GO-CalB				GO-CalB-SrtA		
Temp (°C)	Activity^a^	Selectivity^b^	Time (h)	Activity	Selectivity	Time (h)
4	0.0005	3.3	2	0.0013	10.7	2
4	0.0016		7	0.0019		7
30	0.0025	4.4	2	0.0017	4.5	2
30	0.004		7	0.004		7

a Activity is expressed as micromoles of PUFA (EPA + DHA)/min

b Selectivity is expressed as the ratio between released EPA and released DHA.

## 4 Conclusion

GO nanosheets were functionalized with amine functional groups and applied for the oriented C-terminal attachment of CalB via sortase-mediated immobilization and its functional properties were compared with the same derivative of CalB, randomly immobilized on the same support using glutaraldehyde. Oriented immobilization provides one-step purification and immobilization of CalB directly from the crude extract under mild conditions, resulting in a shortcut in stabilization protocol. This, together with simple production of SrtA in large amounts, makes this method cost-effective. The oriented immobilization produced specific activity higher than the specific activity of the random immobilization, due to the fact that the site-directed attachment allows the enzyme to be uniformly orientated on the surface of GO nanosheets. Surprisingly, the derivative obtained from oriented immobilization exhibited higher thermal stability compared to the results obtained from the randomly immobilized enzyme as well as soluble enzyme. However, the results revealed that both immobilization methods have no a meaningful effect on the co-solvent stability of the immobilized preparations in the three polar organic solvents used. Application of the immobilized preparations for the selective hydrolysis of EPA and DHA from fish oil showed that both GO-CalB and GO-CalB-SrtA could release EPA and DHA in favor of EPA. Furthermore, the immobilized derivatives obtained from oriented immobilization presented almost 2.5 fold higher selectivity compared to the derivative of random immobilization.

## Author contributions

**Faezeh Moosavi:** Investigation; Methodology; Formal Analysis. **Faezeh Ahrari:** Investigation; Formal Analysis. **Gholamreza Ahmadian:** Conceptualization; Resources; Writing - review & editing; Formal analysis. **Mehdi Mohammadi:** Conceptualization; Resources; Writing - original draft; Writing - review & editing.

## Declaration of Competing Interests

The authors declare that they have no known competing financial interests or personal relationships that could have appeared to influence the work reported in this paper.
